# Recurrent atrial fibrillation and stroke in a young adult with congenital heart defects: A case report

**DOI:** 10.1097/MD.0000000000048189

**Published:** 2026-03-27

**Authors:** Kenana Altell, Wasef Alhroub, Maaweya Jabareen, Jinin Frejat, Heba Zatari, Firas Zeedat, Baha Alhadad, Bajis Amr, Zaid Kamel

**Affiliations:** a Faculty of Medicine, Hebron University, Hebron, Palestine; b Cardiology Department, Al Ahli Hospital, Hebron, Palestine.

**Keywords:** atrial fibrillation, atrial septal defect, Ebstein anomaly, ischemic stroke

## Abstract

**Rationale:**

Atrial septal defect (ASD) is a common congenital heart defect that can lead to left-to-right shunting, volume overload, and subsequent right atrial dilation – conditions that predispose to atrial arrhythmias such as atrial fibrillation (AF). The coexistence of Ebstein anomaly further increases the risk of early-onset arrhythmias and thromboembolic events. Stroke in young patients with such congenital abnormalities remains a rare but serious complication.

**Patient concerns:**

An 18-year-old female presented with intermittent palpitations and exertional chest discomfort. She had a past medical history of AF, Ebstein anomaly, repaired ASD, and a previous ischemic stroke. Despite early surgical interventions, including ASD closure and tricuspid valve plasty, she continued to experience arrhythmias.

**Diagnoses:**

Echocardiography showed mildly reduced left ventricular function with an ejection fraction of 45%, and electrocardiogram revealed rapid AF and right bundle branch block. Her history, clinical findings, and past echocardiograms confirmed recurrent AF associated with congenital structural heart defects, including ASD and Ebstein anomaly.

**Interventions:**

Management included rhythm monitoring, supportive care, and lifestyle modifications. A prior stroke prompted consideration of long-term anticoagulation and ongoing cardiac monitoring to reduce thromboembolic risk. Antiarrhythmic therapy and follow-up imaging were used to assess cardiac function and arrhythmic burden.

**Outcomes:**

The patient showed clinical stability on follow-up, with improved ejection fraction and no recurrence of stroke. Serial electrocardiograms and echocardiograms over 2 and a half years revealed no major complications, although arrhythmic risk remained elevated.

**Lessons:**

This case underscores the importance of long-term follow-up in patients with congenital heart disease, even after surgical correction. The coexistence of ASD and Ebstein anomaly can predispose to early-onset AF and stroke. Early detection and individualized management strategies, including rhythm control and anticoagulation, are critical in reducing morbidity and preventing recurrence.

## 1. Introduction

Atrial septal defects (ASD) are among the most common congenital heart defects. The prevalence of these defects is 9% to 13% of all congenital cardiac defects.^[1]^ The morphological defect in the atrial septum will, in most cases, lead to a left-to-right shunt because of higher pressure in the left atrium. The shunt results in an increased volume load on the right side of the heart. By the time of diagnosis, several other morphological changes in the heart are frequently found. Right ventricular dilatation and right atrial enlargement are among the most common changes.^[1,2]^ Its natural history can be associated with the development of arrhythmias, right heart failure, stroke, and pulmonary hypertension.^[3]^

However, the main cause of morbidity and mortality in ASD patients is attributed to the development of atrial tachyarrhythmias. Volume overload secondary to left-to-right atrial shunt leads to atrial structural remodeling, which is more pronounced in the right atrium and frequently results in supraventricular arrhythmias, especially atrial flutter and atrial fibrillation (AF).^[4]^ It is noteworthy that the incidence of these complications increases with age; 14% of patients were between 20 and 40 years of age, 24% of patients between 40 and 60 years, and 100% over 60 years of age.^[3]^

While ASD is a common congenital defect, the combination of ASD and Ebstein anomaly, leading to the subsequent development of atrial arrhythmias such as AF and atrial flutter, in a young patient is relatively rare. This case report presents an 18-year-old female patient with a history of both ASD repair and Ebstein anomaly, who later developed recurrent atrial flutter and AF. Despite early surgical intervention, the combination of Ebstein anomaly and ASD contributed to the early onset of arrhythmias.

## 2. Case presentation

An 18-year-old female with a history of AF, Ebstein anomaly of the tricuspid valve, ASD, left ventricular dysfunction, and total anomalous pulmonary venous connection presented with intermittent palpitations and exertional chest discomfort over the past 2 months. During early childhood, the patient was diagnosed with ASD and Ebstein anomaly and underwent multiple staged surgical interventions, including ASD closure, tricuspid valve plasty, Glenn anastomosis, and repair of Ebstein anomaly.

At the age of 15, the patient had an ischemic stroke that was treated with anticoagulation, with rapid AF that required cardioversion; in addition, she was diagnosed with epilepsy. A previous echocardiogram performed a year ago revealed severe left ventricular dilatation with an ejection fraction of 24%. Previous electrocardiograms (ECGs) documented episodes of rapid AF, necessitating cardioversion. At the time, she presented with intermittent palpitations and chest discomfort on exertion.

**Figure 1.**
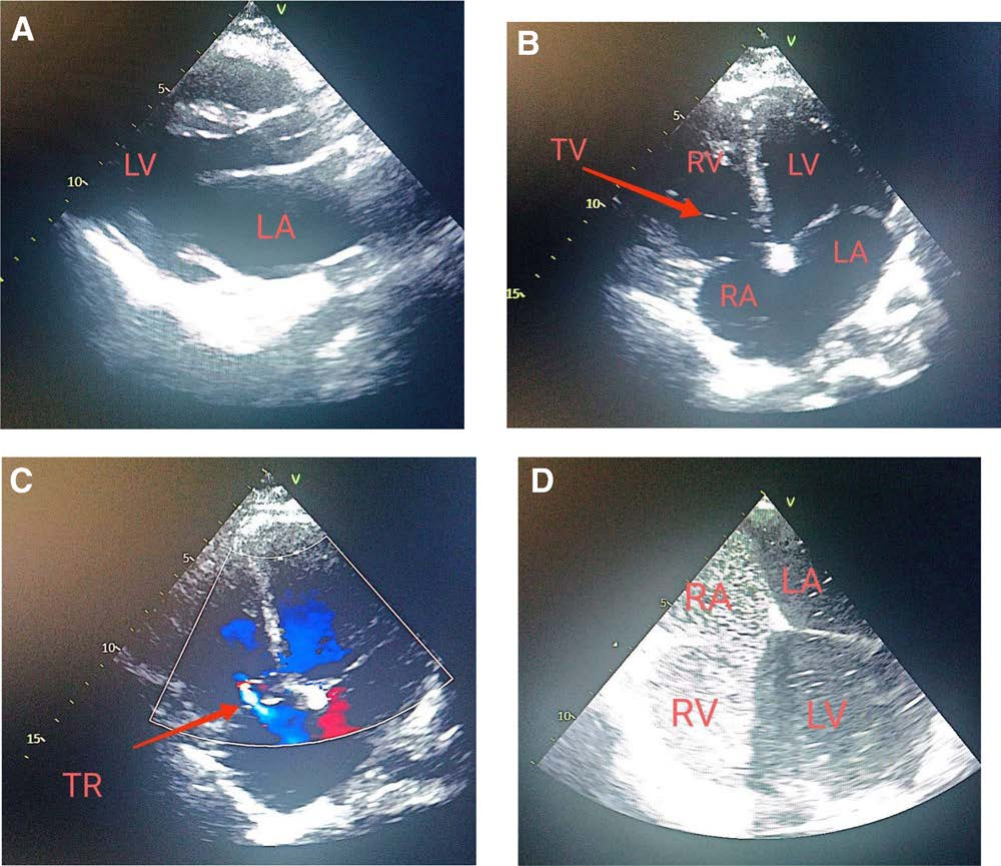
(A) TTE in the parasternal long-axis view showing a mildly dilated LA with preserved left ventricular structure. (B) TTE with apical 4 chambers view showing mildly dilated both atria, and mildly dilated RV. (C) TTE with apical 4 chambers view showing mild TR. (D) TEE showing positive bubble test (during Valsalva maneuver) due to residual shunt post ASD closure. ASD = atrial septal defect, LA = left atrium, LV = left ventricle, RA = right atrium, RV = right ventricle, TEE = transesophageal echocardiogram, TR = tricuspid regurgitation, TTE = transthoracic echocardiogram, TV = tricuspid valve.

**Figure 2.**
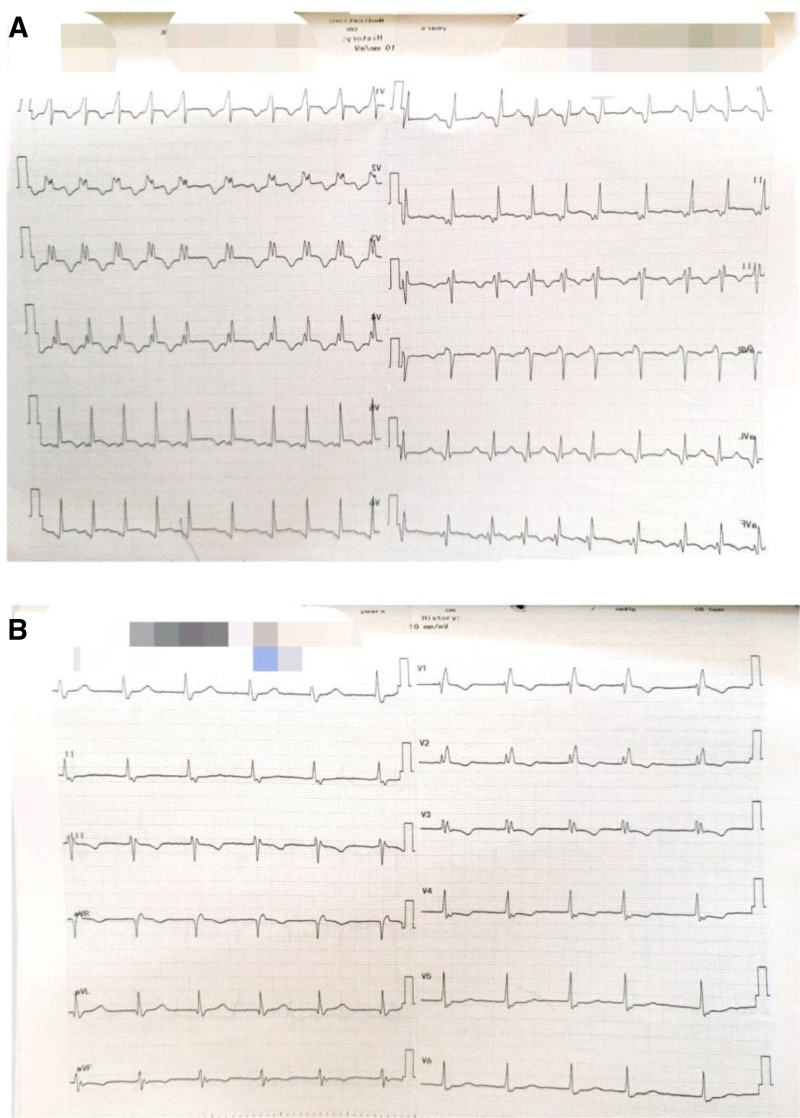
(A) Twelve-lead ECG showing controlled atrial fibrillation with a ventricular rate of ~120 bpm and RBBB, marked by absent P waves, irregular RR intervals, a widened QRS complex (>120 ms), rSR’ in V1, and broad S waves in I and V6. (B) Twelve-lead ECG showing controlled atrial fibrillation with a ventricular rate of 70 bpm and RBBB, characterized by irregular RR intervals, absent P waves, and a widened QRS complex with an rSR’ pattern in V1. ECG = electrocardiogram, RBBB = right bundle branch block.

On examination, she appeared clinically stable with an oxygen saturation of 96% on room air, without signs of cyanosis, edema, or residual ASD. Repeat echocardiography showed a normal-sized left ventricle with mildly reduced systolic function and an ejection fraction of 45%, as shown in Figure 1. ECG findings included irregular heart rate, rapid AF, and right bundle branch block, as shown in Figure 2.

Laboratory investigations were largely unremarkable, with a normal metabolic panel and negative cardiac biomarkers. A chest radiograph was normal. Additional findings included an international normalized ratio of 1.26 and an elevated activated partial thromboplastin time of 59.2 seconds. Lipid panel results showed total cholesterol of 112 mg/dL, low-density lipoprotein of 55 mg/dL, and high-density lipoprotein of 46 mg/dL.

Her management plan includes regular monitoring of left ventricular function and arrhythmias, along with lifestyle modifications to reduce cardiac strain. A 6-month follow-up, including serial ECGs and echocardiograms, revealed no significant complications. Future management will focus on addressing arrhythmias and thrombotic risks as needed.

## 3. Discussion

ASD is a common congenital heart disease that occurs in 25% of the pediatric patient population.^[5]^ It results from the imperfect closure of the communication between the right and left atria, which may involve defects in the septal membrane or other abnormalities facilitating interatrial communication. ASD is classified into 5 subtypes, ordered from the most to the least common: patent foramen ovale, ostium secundum defect, ostium primum defect, sinus venosus defect, and coronary sinus defect. Small ASDs often close spontaneously during childhood; however, larger defects that do not close on their own may necessitate percutaneous or surgical intervention to minimize the risk of associated complications such as stroke, arrhythmias, and pulmonary hypertension.^[6]^

ASD can contribute to a paradoxical embolism as a complication arising from the anatomical understanding of this pathology, through the abnormal communication between the right and left atria, termed as right-to-left shunt, which increases the risk of stroke. In a normal, healthy heart, blood flows from the right atrium into the right ventricle and is pumped to the lungs for oxygenation, and it flows back to the left side of the heart. However, in the presence of an ASD, a passage allows blood to flow from the right atrium into the left atrium directly, bypassing the lungs.^[7]^ This can create a pathway for a thrombus (clot) formed in the venous circulation to travel through the heart. From there, the clot can enter systemic circulation and potentially lodge in the cerebral arteries, causing a transient ischemic attack or an established stroke in worst scenario. This occurrence is termed a paradoxical embolism because, under normal circumstances, venous clots should only affect the lungs. The presence of an ASD, however, allows them to bypass the pulmonary filter and reach the brain or other organs.^[8]^

Ebstein anomaly is a congenital heart defect where the tricuspid valve leaflets are displaced into the right ventricle, causing right ventricular dysfunction and tricuspid regurgitation. It can result from lithium exposure in utero and often leads to arrhythmias like AF because of impaired blood flow, right atrial dilation, and increased pressure.^[9]^ These changes create a good environment for arrhythmias, with the dilated atrium and altered conduction pathways. Symptoms range from asymptomatic to severe, influenced by factors like tricuspid valve displacement, regurgitation, right ventricular function, and associated defects (e.g., ASD, pulmonary valve stenosis). Common symptoms include cyanosis, heart failure, arrhythmias, and exertional dyspnea. Surgical intervention is often needed for management.^[10]^

AF is the most prevalent sustained arrhythmia, characterized by disorganized, rapid, and irregular atrial electrical activity, which results in an irregular ventricular response, and it can be classified into various subtypes based on its duration and clinical characteristics. This disruption in normal atrial function leads to a loss of atrial contractility, impairing the complete evacuation of blood from the atrial appendage and thereby increasing the risk of thrombus formation and subsequent thromboembolic events.^[11]^ Even with improvements in treatment, recurrence rates remain high, especially in persistent AF, hence the need for an understanding of the underlying mechanisms. DNA methylation, histone modifications, chromatin remodeling, RNA methylation, and noncoding RNAs are all examples of epigenetic regulation, which plays an important role in the structural, electrical, and inflammatory remodeling that underlies AF. Electrical remodeling linked to AF results in cellular electrophysiological malfunction and the continuation of arrhythmias. There is growing evidence that ion channel expression is regulated by epigenetic processes. Histone deacetylases deacetylate histone proteins to regulate gene expression.^[12]^

In an 18-year-old female with Ebstein anomaly, ASD, and rapid AF, the pathogenesis of stroke can involve several mechanisms, including paradoxical embolism, intracardiac thrombus formation, and cerebral hypoperfusion. Rapid AF in the context of Ebstein anomaly and ASD can lead to impaired atrial contraction, blood stasis within the atrium, and turbulent blood flow, which promotes thrombus formation, particularly in the left atrial appendage or right atrium. These thrombi may dislodge and pass through the ASD, entering the left atrium and causing embolic events in the brain, leading to transient ischemic attack or stroke.^[13]^ In addition, the abnormal communication between the right and left atria in Ebstein anomaly or ASD can allow venous thrombi, typically from the lower extremities, to bypass the lungs and enter the systemic circulation. These emboli can pass through the ASD into the left atrium and subsequently reach the cerebral circulation, causing a stroke.^[14]^ Furthermore, the irregular and rapid ventricular response associated with AF can result in hemodynamic instability, leading to periods of inadequate blood flow to the brain (cerebral hypoperfusion), which may contribute to ischemic injury in the cerebral vasculature, further increasing the risk of stroke.^[15]^

Similar cases have been documented in the literature. For instance, a 28-year-old woman with Ebstein anomaly and an ASD presented with palpitations and tachycardia; surgical interventions targeting the arrhythmias resulted in favorable outcomes,^[16]^ differing from the present case by the older age at presentation and the absence of stroke-related complications. Another report described a 15-year-old female with complex congenital heart defects, including Ebstein anomaly and ASD, who presented with dyspnea and hemoptysis, highlighting the coexistence of cardiac malformations and pulmonary manifestation.^[17]^

Diagnosing the cause of stroke in patients with multiple abnormalities is a complex process, as factors like structural heart defects and arrhythmias can independently increase stroke risk through mechanisms such as embolism or altered blood flow. Imaging tools play a crucial role in confirming underlying conditions and pinpointing the precise cause. Continuous cardiac monitoring, such as Holter monitoring, is vital to assess AF’s role in thrombus formation,^[18]^ and coagulation profile testing helps identify any additional stroke risk factors, including prothrombotic conditions, given the patient’s age and gender.^[19]^

Cases like this, complicated by an acute stroke, require detailed management of both the stroke and the underlying cardiac issues. In the acute setting, rapid assessment through computed tomography or magnetic resonance imaging is key to distinguishing between ischemic and hemorrhagic stroke. If ischemic stroke is confirmed, reperfusion therapies like tissue plasminogen activator or thrombectomy may be considered. However, the patient’s congenital heart defects and AF complicate treatment due to increased thromboembolism risk.^[20]^

Managing AF during the acute stroke phase can be challenging, as rapid AF, especially in the setting of ASD or Ebstein anomaly, can exacerbate the risk of embolism, and the choice of anticoagulation or antiplatelet therapy must be made cautiously. Early anticoagulation therapy after stroke could increase the risk of bleeding, particularly in patients who have had a recent ischemic event, thus requiring careful monitoring.^[21]^ For this patient, rapid rhythm control to stabilize AF is also important, which could involve medications such as β-blockers, with careful attention to heart rate and rhythm.

While adult AF guidelines recommend anticoagulation (e.g., warfarin) based on risk stratification scores, these tools have not been validated in pediatric patients with congenital heart disease-related AF. In children, long-term anticoagulation is therefore typically managed with carefully monitored vitamin K antagonists or low-molecular-weight heparin, as evidence for the safety and efficacy of direct oral anticoagulants in this population remains limited.^[22]^ The American Heart Association emphasizes an individualized approach, highlighting that thrombosis prevention and treatment in pediatric congenital heart disease should rely on expert-driven protocols rather than standardized adult-based scoring systems.^[23]^

In patients with Ebstein anomaly and atrial arrhythmias, catheter ablation is feasible but technically challenging, with modest long-term success and higher efficacy for cavotricuspid isthmus-dependent flutter than for AF; repeat procedures are often required, and ablation is best reserved for drug-refractory cases in specialized Adult Congenital Heart Disease-Electrophysiology centers.^[24]^ Surgical ablation (Maze/lesion sets) procedures reduce arrhythmias when added during tricuspid surgery; however, it is usually considered concomitantly with an indicated structural operation (e.g., valve repair) rather than as a stand-alone redo in stable patients.^[25]^ For this patient, guideline-directed anticoagulation and medical rhythm/rate control were prioritized, deferring invasive options unless recurrences persist or future surgery is indicated.

Recent mechanistic studies have begun to elucidate how chronic atrial stretch and remodeling in patients with ASD and Ebstein anomaly create a profibrotic substrate that fosters atrial tachyarrhythmias. Volume overload from residual shunting and tricuspid regurgitation induces stretch-activated signaling in atrial fibroblasts, triggering collagen deposition and reactive fibrosis, which in turn produces conduction slowing and heterogeneous refractoriness conducive to reentrant circuits. Concurrently, oxidative stress and inflammatory cytokine release amplify fibroblast activation, while altered connexin expression at myocyte–myocyte junctions disrupts electrical coupling.^[26]^ Together, these processes form a self-reinforcing loop of structural and electrical remodeling that underpins the persistence and recurrence of AF in this population.

This case is unique because it presents a very young patient with a rare combination of congenital heart defects, ASD, Ebstein anomaly, and total anomalous pulmonary venous connection, who developed recurrent AF and an ischemic stroke despite early surgical repair. Unlike most reported cases where arrhythmias and thromboembolic complications typically appear later in adulthood, this patient experienced both stroke and recurrent atrial arrhythmias during adolescence, along with secondary complications such as epilepsy. The coexistence of multiple structural anomalies, early onset of AF, and stroke in a teenager highlights the complex interplay between congenital defects, arrhythmogenic substrates, and thromboembolic risk, underscoring the importance of long-term surveillance and individualized management even after early corrective surgery.

This case offers new insights into the long-term sequelae of congenital heart defects, particularly in young patients with complex anomalies such as ASD and Ebstein anomaly, and underscores the need for nuanced risk stratification beyond standard scoring systems like CHA_2_DS_2_-VASc. A notable clinical lesson is the persistence of arrhythmias and thromboembolic risk despite early surgical correction, suggesting that structural correction alone may not fully mitigate long-term electrophysiological consequences.

## 4. Conclusion

This case highlights the need for a multidisciplinary approach when managing young patients with congenital heart defects and acute stroke, emphasizing the balance between thromboembolic risk and bleeding complications. It also underscores the importance of considering all of the abnormalities when selecting treatment strategies. Early diagnosis through imaging and ongoing monitoring is vital for effective management. Treatment should carefully weigh the risks of anticoagulation, control of arrhythmias, and possible surgical options. Long-term follow-up and a collaborative approach are essential for improving outcomes and preventing future strokes.

Future research should focus on personalized management for such cases, including tailored anticoagulation, optimal timing for interventions, and better arrhythmia management. In addition, exploring long-term outcomes and genetic factors may further refine clinical practice.

## Acknowledgments

We would like to thank the patient’s family for cooperating in this study.

## Author contributions

**Conceptualization:** Firas Zeedat.

**Supervision:** Baha Alhadad, Bajis Amr, Zaid Kamel.

**Validation:** Jinin Frejat.

**Visualization:** Heba Zatari.

**Writing – original draft:** Kenana Altell, Maaweya Jabareen, Jinin Frejat, Heba Zatari.

**Writing – review & editing:** Wasef Alhroub.
